# Dual-Site aiTBS for Suicidal Ideation in Adolescents With Major Depressive Disorder: A Randomized Clinical Trial

**DOI:** 10.1001/jamanetworkopen.2026.13178

**Published:** 2026-05-19

**Authors:** Dong Huang, Rongxu Zhang, Shunkai Lai, Xiaojie Ye, Munila Abula, Xiaodong Song, Peiying Cao, Yiliang Zhang, Jianzhao Zhang, Shuming Zhong, Yanbin Jia

**Affiliations:** 1 Department of Psychiatry, First Affiliated Hospital of Jinan University, Guangzhou, China

## Abstract

**Importance:**

Accelerated intermittent theta burst stimulation (aiTBS) is an effective rapid-acting treatment for suicidal ideation in adults with major depressive disorder (MDD). However, evidence in adolescents remains limited, and whether dual-site frontal-cerebellar aiTBS enhances antisuicidal efficacy in this population is unknown.

**Objective:**

To investigate the clinical effectiveness and safety of dual-site aiTBS targeting the left dorsolateral prefrontal cortex (DLPFC) and cerebellum vs single-site DLPFC aiTBS for reducing suicidal ideation in adolescents with MDD.

**Design, Setting, and Participants:**

This double-blind, randomized clinical trial with a 1-month follow-up was conducted at the First Affiliated Hospital of Jinan University (Guangzhou, China) from September 2023 to May 2025. Adolescents (aged 12-18 years) with MDD and suicidal ideation and without comorbid medical or neurologic conditions were enrolled and randomly assigned to dual-site aiTBS or single-site aiTBS.

**Interventions:**

Patients received 5 daily sessions of aiTBS over 4 days. Dual-site aiTBS delivered active stimulation to both the left DLPFC and left cerebellum, while single-site aiTBS delivered active stimulation to the left DLPFC with sham stimulation to the left cerebellum.

**Main Outcome and Measure:**

The primary outcome was the change from baseline to day 4 in the Beck Scale for Suicide Ideation (BSI) score (range, 0-38; higher scores indicate greater severity of suicidal ideation), assessed with a linear mixed-effects model.

**Results:**

Of 79 adolescents assessed for eligibility, a total of 59 were included (45 females [76%]; mean [SD] age, 14.78 [1.72] years). Of these, 29 were randomized to the dual-site aiTBS group (23 [79%] female) and 30 to the single-site group (22 [73%] female). For the primary outcome, patients receiving dual-site aiTBS showed a significantly greater reduction in mean BSI scores compared with those receiving single-site aiTBS at day 4 (difference, 4.94 points [95% CI, 0.73-9.14 points]; *t* = 2.35; *P* = .02; Cohen *d*, 0.61 [95% CI, 0.09-1.13]). No serious adverse events in either group were observed.

**Conclusions and Relevance:**

This randomized clinical trial found that dual-site aiTBS targeting the left DLPFC and cerebellum was more effective than single-site left DLPFC aiTBS in reducing suicidal ideation for adolescents with MDD and was well tolerated. The findings suggest this protocol may be a promising strategy for rapid reduction of suicidal ideation in the adolescent population.

**Trial Registration:**

Chinese Clinical Trial Registry Identifier: ChiCTR2300068954

## Key Points

**Question:**

Is dual-site accelerated intermittent theta burst stimulation (aiTBS) targeting the left dorsolateral prefrontal cortex (DLPFC) and cerebellum more effective than single-site DLPFC stimulation in alleviating suicidal ideation among adolescents with major depressive disorder (MDD)?

**Findings:**

In this randomized clinical trial of 59 adolescents aged 12 to 18 years with MDD and suicidal ideation, dual-site aiTBS significantly reduced suicidal ideation after the 4-day treatment compared with single-site stimulation.

**Meaning:**

The findings indicate that dual-site aiTBS targeting both the DLPFC and cerebellum may provide a rapid and effective treatment option for suicidal ideation in adolescents with MDD.

## Teaser

This randomized clinical trial compares the efficacy of dual-site accelerated intermittent theta burst stimulation (aiTBS) to the left dorsolateral prefrontal cortex (DLPFC) and cerebellum vs to the DLPFC alone for reducing suicidal ideation in adolescents with major depressive disorder in China.

## Introduction

Adolescent suicide is a global public health concern, primarily driven by major depressive disorder (MDD).^1^ Among adolescents with MDD in the US, rates of suicidal ideation and attempts have increased to 60.0% and 30.0%, respectively, compared with 13.1% and 6.0% in the overall population.^2,3^ Suicidal ideation is a well-established predictor of suicide attempts, and both are critical risk factors for death by suicide.^4^ Thus, developing and implementing interventions that can rapidly reduce suicidal ideation are a critical priority for suicide prevention in adolescents with MDD.

Accelerated intermittent theta burst stimulation (aiTBS), such as Stanford neuromodulation therapy (SNT), confers rapid antisuicidal effects, making it preferable to standard iTBS for adults with MDD at high suicide risk.^5,6^ However, whether this approach yields similar benefits in adolescents with MDD has not been established. Following reports of rapid antisuicidal effects with a 20-session aiTBS regimen (5 daily sessions over 4 days) in adults with MDD,^7,8,9^ our group adapted this protocol for adolescents with depression by targeting both the left dorsolateral prefrontal cortex (DLPFC) and left cerebellum.^10^ The intervention was well tolerated, had high adherence, and significantly reduced suicidal ideation, indicating the feasibility and efficacy of this dual-site approach in adolescents with MDD. Its abbreviated 20-session schedule may also offer superior patient acceptability over the more intensive 50-session SNT.

The cerebellum represents a promising dual-site aiTBS target for MDD, based on its unique neurobiology (high neuronal density and plasticity)^11,12^ and its integral role in depression-relevant prefrontal-cerebellar circuits.^13,14,15^ Furthermore, converging evidence has implicated the cerebellum in suicide risk, documenting structural, functional, and metabolic alterations in suicidal patients with depression, including adolescents.^16,17,18,19,20,21,22^ Our group previously identified irregular dynamic functional connectivity in the left cerebellum as a predictor of suicidal ideation severity.^23^ Additionally, aberrant cerebellum-DLPFC connectivity predicted response to DLPFC-targeted repetitive transcranial magnetic stimulation (rTMS) in MDD.^24^ Collectively, these findings support that dual-site aiTBS to the left DLPFC and cerebellum may represent a novel, mechanistically grounded intervention for suicidal ideation in adolescents with MDD.

We performed a randomized clinical trial to assess the efficacy and safety of dual-site aiTBS (targeting both the left DLPFC and left cerebellum) compared with standard single-site aiTBS (targeting the left DLPFC alone) in rapidly reducing suicidal ideation among adolescents with MDD. We hypothesized that dual-site vs single-site aiTBS would yield a significantly greater reduction in Beck Scale for Suicide Ideation (BSI) scores after 20 sessions.

## Methods

This randomized, double-blind, sham-controlled clinical trial was conducted at the First Affiliated Hospital of Jinan University in Guangzhou, China, from September 2023 to May 2025. The study protocol is available in Supplement 1. The hospital’s institutional ethics committee approved the study, and written informed consent was obtained from all participants and their legal guardians, in line with the Declaration of Helsinki.^25^ This trial was reported in accordance with the Consolidated Standards of Reporting Trials (CONSORT) reporting guideline for randomized clinical trials.

### Participants

Participants were recruited through physician referrals and posted flyers. The inclusion criteria were (1) diagnosis of MDD by 2 independent psychiatrists using the *Diagnostic and Statistical Manual of Mental Disorders* (Fifth Edition), (2) age of 12 to 18 years, (3) 24-item Hamilton Depression Rating Scale (HDRS-24) score higher than 20 on a scale from 0 to 76 (higher scores indicate more severe symptoms), (4) BSI score of 12 or higher on a scale from 0 to 38 (higher scores indicate more severe symptoms), and (5) Young Mania Rating Scale (YMRS) score lower than 7 on a scale from 0 to 60 (higher scores indicate more severe symptoms).

Exclusion criteria were (1) comorbid medical or neurologic conditions (eg, epilepsy, brain tumors, or hyperthyroidism); (2) history of rTMS, transcranial direct current stimulation, or electroconvulsive therapy within the past 3 months; and (3) contraindications such as metal implants. Criteria for study withdrawal were (1) nonadherence, defined as refusal of the assigned treatment on 2 or more occasions; (2) intolerance due to serious adverse effects; (3) clinical deterioration requiring a fundamental change in treatment strategy; or (4) protocol violations, including changes to a concomitant medication type or dose during the 4-day intervention.

### Randomization and Blinding

Simple randomization (1:1) assigned participants to dual- or single-site treatment using a computer-generated random sequence prepared by an independent investigator (J.Z.) with no involvement in outcome assessment. Allocation was concealed in sequentially numbered opaque envelopes and revealed only at the moment of intervention to an operator (including D.H.), who was excluded from all assessments. Participants, assessors (including R.Z.), and clinical staff were blinded to treatment assignments. Blinding integrity was evaluated postintervention via participants’ allocation guesses.

### Targeting and Procedure

All operators completed standardized aiTBS training before study initiation. aiTBS was administered with a MagStim Rapid^2^ transcranial magnetic stimulator (Magstim Company Ltd) using a 70-mm air-cooled figure-of-8 coil. The DLPFC was identified with the 5-cm rule, and the cerebellar target was located 3 cm lateral to and 1 cm inferior to the inion.^26^ Resting motor threshold (RMT) was defined as the minimum stimulation intensity that elicited a visible twitch in the relaxed right abductor pollicis brevis muscle in at least 5 of 10 consecutive trials. Each individual iTBS session delivered 600 pulses over 20 cycles of 2-second 20-Hz triple-pulse bursts with an 8-second intertrain interval. The aiTBS protocol comprised 5 of these 600-pulse iTBS sessions per day at 1-hour intervals over 4 days (12 000 total pulses). Sham stimulation was applied with the coil tilted 90° to the scalp, producing noise and vibratory sensations similar to real stimulation. The dual-site group received sequential active aiTBS on the left DLPFC followed by the left cerebellum, whereas the single-site group received active aiTBS on the left DLPFC followed by sham stimulation on the left cerebellum. Stimulation intensity was set at 80% of the RMT.

### Clinical Assessments and Outcomes

Clinical symptom assessments were performed at baseline and daily over the 4-day intervention (using both clinician-rated and self-report scales) and at the 1-month follow-up (self-report scales only). The HDRS-24 served as a screening tool. The YMRS scores were evaluated for mood switches emerging from treatment, characterized by a score of 12 or higher.^27^ The primary outcome was the change in BSI scores from baseline to day 4 (postintervention).

Secondary outcomes included (1) the daily change from baseline in scores on all outcome measures over the 4-day intervention (BSI, Beck Depression Inventory [BDI; score range, 0-63], Beck Hopelessness Scale [BHS; score range, 0-20], Columbia-Suicide Severity Rating Scale [C-SSRS; score range, 0-25], Montgomery-Åsberg Depression Rating Scale [MADRS; score range 0-60], and Hamilton Anxiety Rating Scale [HAMA; score range, 0-56], with higher scores on all measures indicating more severe symptoms); (2) the change in scores of the self-report scales (BSI, BDI, and BHS) from baseline to month 1; (3) response rate at day 4, defined as a reduction of 50% or more in BSI scores for suicidal ideation or MADRS scores for depression; (4) remission rates at day 4, defined as BSI score of 8 or lower for suicidal ideation^28^ or MADRS score of 10 or lower for depression^29^; (5) correlation between changes in BSI scores and BDI or BHS scores from baseline to day 4 and to month 1; and (6) incidence of adverse events during the 4-day intervention period (eMethods in Supplement 2).

### Sample Size Calculation

The sample size calculation was informed by pilot data, which showed a between-group difference in BSI scores, supporting a 5-point margin for this trial. With 80% power and a 2-sided α of .05, the calculation required 48 participants (24 per group). The target enrollment was set at 58 participants (29 per group) to account for a 20% dropout rate.

### Statistical Analysis

Statistical analyses were performed using IBM SPSS, version 25 (IBM Corp). Intention-to-treat analysis was conducted, with missing data estimated by mean interpolation. Two-sided *P* < .05 was deemed statistically significant. The Shapiro-Wilk test was used to evaluate normality. Baseline demographic and clinical characteristics were analyzed between groups using χ^2^ tests for categorical variables and either independent-sample *t* tests or Mann-Whitney *U* tests for continuous variables.

For the primary outcome and all continuous secondary outcomes, a linear mixed-effects model was used to assess treatment effects (between-group differences in mean score changes). The model incorporated fixed effects for group, time, and their interaction, along with a random intercept for participants. The unstructured covariance structure was selected based on the minimum Akaike information criterion. The robustness of the antisuicidal and antidepressant effects was evaluated in sensitivity analyses using linear mixed models on the C-SSRS and BDI scores, respectively. The χ^2^ test or Fisher exact test was used to compare response and remission rates, as well as the incidence of adverse events, between groups. The Pearson correlation coefficient was used for correlational analyses.

## Results

### Participants

**Figure 1.**
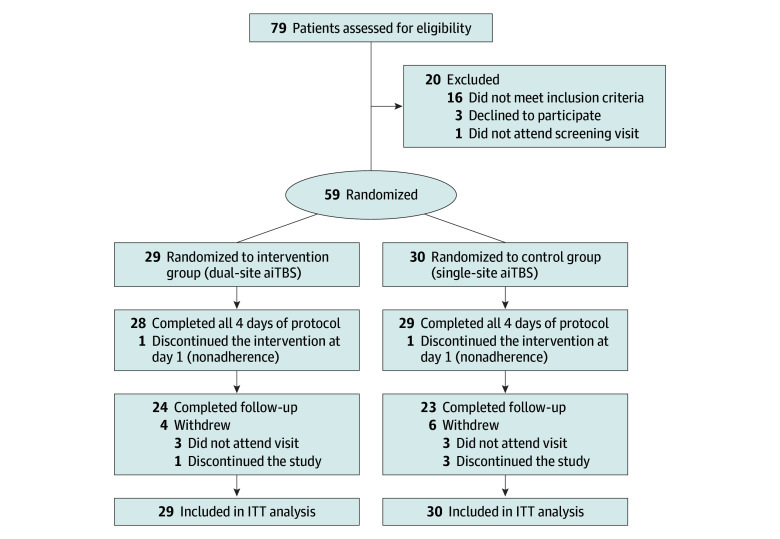
CONSORT Flow Diagram aiTBS indicates accelerated intermittent theta burst stimulation; CONSORT, Consolidated Standards of Reporting Trials; ITT, intention to treat.

Of 79 adolescents assessed for eligibility, a total of 59 eligible patients (45 females [76%] and 14 males [24%]; mean [SD] age, 14.78 [1.72] years) were recruited (Figure 1). Of these, 29 were randomized to the dual-site group (23 females [79%] and 6 males [21%]; mean [SD] age, 14.86 [1.73] years) and 30 to the single-site group (22 females [73%] and 8 males [27%]; mean [SD] age, 14.70 [1.75] years). There was no significant difference in baseline demographic and clinical variables between the dual- and single-site groups (Table 1). Withdrawals included 2 participants (1 per group) during the 4-day intervention and 10 (4 [14%] dual-site vs 6 [20%] single-site) during follow-up, with no significant between-group difference (χ^2^ = 0.34; *P* = .56). Correct group allocation was guessed by 14 of 29 participants (48%) in the dual-site group and 19 of 30 (63%) in the single-site group (χ^2^ = 1.36; *P* = .24).

**Table 1.** Demographic and Clinical Characteristics of the Randomized Patients

Characteristic	Patients (n = 59)^a^	
	Dual-site (n = 29)	Single-site (n = 30)
Sex		
Female	23 (79)	22 (73)
Male	6 (21)	8 (27)
Age, mean (SD) y	14.86 (1.73)	14.70 (1.75)
Educational level, mean (SD) y	8.59 (1.68)	8.37 (1.88)
Place of residence		
Urban	7 (24)	8 (27)
Rural	22 (76)	22 (73)
BMI, mean (SD)	20.48 (3.74)	20.31 (4.44)
Family history of psychiatric disorders	4 (14)	3 (10)
History of suicide attempts	20 (69)	19 (63)
History of self-injury	22 (76)	24 (80)
Total duration of MDD, median (IQR), y	2.00 (0.83-3.00 )	2.25 (1.00-3.25 )
Duration of current depressive episode, median (IQR), mo	3.00 (2.00-6.00 )	2.50 (1.00-15.00 )
Duration of current suicidal episode, median (IQR), mo	2.00 (0.63-3.00 )	1.00 (1.00-3.13 )
Total depressive episodes, median (IQR), No.	1.00 (1.00-2.00)	1.00 (1.00-2.00)
Age at MDD onset, median (IQR), y	13.00 (12.00-14.00)	12.00 (11.00-13.00)
Medication stratification		
Antidepressant	10 (36)	13 (43)
Mood stabilizer	22 (76)	19 (63)
Atypical antipsychotic	20 (69)	16 (53)
Baseline score, mean (SD)		
Depression (MADRS)^b^	30.38 (4.75)	30.43 (4.62)
Suicide ideation (BSI)^c^	22.55 (3.86)	23.33 (3.63)

Abbreviations: BMI, body mass index (calculated as weight in kilograms divided by height in meters squared); BSI, Beck Scale for Suicide Ideation; MADRS, Montgomery-Åsberg Depression Rating Scale.

^a^ Variables are reported as number (percentage) of patients unless otherwise indicated.

^b^ MADRS score range, 0 to 60, with higher scores indicating more severe symptoms.

^c^ BSI score range, 0 to 38, with higher scores indicating more severe symptoms.

### Primary Outcome

**Figure 2.**
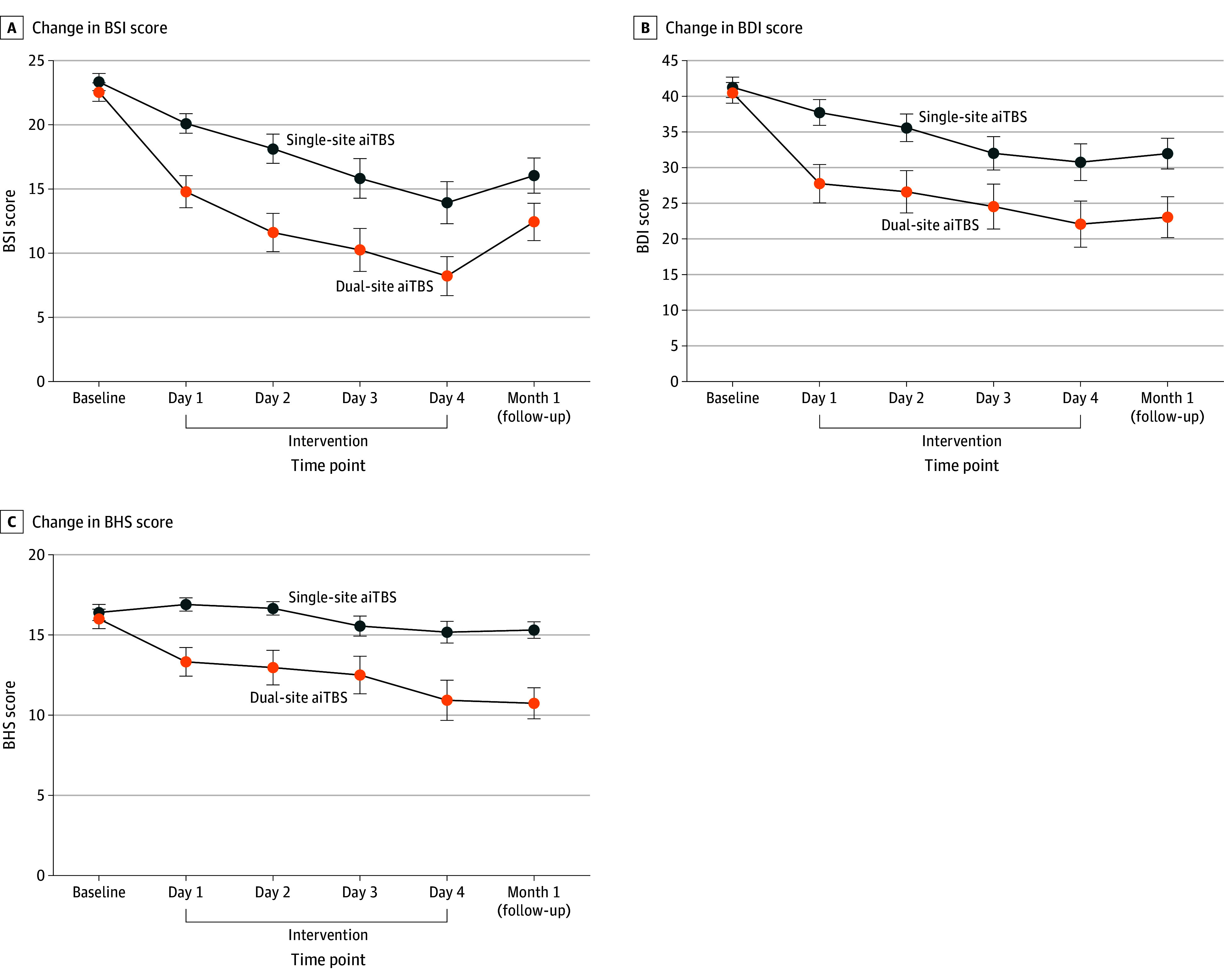
Line Graphs Showing Clinical Outcomes of the Accelerated Intermittent Theta Burst Stimulation (aiTBS) Treatment Data markers denote means; error bars, SEs. BDI indicates Beck Depression Inventory (score range, 0-63); BHS, Beck Hopelessness Scale (score range, 0-20); and BSI, Beck Scale for Suicide Ideation (score range, 0-38), with higher scores on all 3 measures indicating more severe symptoms.

For the primary outcome (change in BSI scores at day 4), analysis using a linear mixed-effects model indicated significant effects of group (*F*_1,56_ = 4.75; *P* = .03), time (*F*_1,54_ = 119.13; *P* < .001), and time × group interaction (*F*_1,54_ = 4.46; *P* = .04) for BSI scores. A post hoc test showed that the dual-site group had a significantly greater reduction in mean BSI scores at day 4 than the single-site group (difference, 4.94 points [95% CI, 0.73-9.14 points]; *t* = 2.35; *P* = .02; Cohen *d*, 0.61 [95% CI, 0.09-1.13]) (Figure 2A). The mean (SD) BSI score decreased from 22.55 (3.86) to 8.21 (8.19) points in the dual-site group (mean [SD] reduction, 14.34 [7.88] points) and from 23.33 (3.63) to 13.93 (8.98) points in the single-site group (mean [SD] reduction, 9.40 [8.23] points) (Table 2). The significant group main effect remained robust after adjusting for baseline MADRS (*F*_1,56_ = 5.26; *P* = .03) and BHS (*F*_1,57_ = 4.45; *P* = .04) scores.

**Table 2.** Primary and Secondary Outcomes

Outcome^a^	Patient group, mean (SD)		Single-site vs dual-site group		
	Dual-site (n = 29)	Single-site (n = 30)	Least squares mean difference (95% CI)^b^	Time × group interaction^c^	*P* value
**Primary outcome**					
Change in BSI score, baseline to day 4^d^	14.34 (7.88)	9.40 (8.23)	3.10 (0.25 to 5.96)	*F*_1,54_ = 4.46	.04
**Secondary outcomes**					
BSI score^d^					
Baseline	22.55 (3.86)	23.33 (3.63)	4.66 (1.53 to 7.78)	*F*_4,55_ = 3.08	.02
Day 1	14.79 (6.72)	20.10 (4.18)			
Day 2	11.61 (8.01)	18.14 (6.22)			
Day 3	10.25 (8.98)	15.83 (8.46)			
Day 4	8.21 (8.19)	13.93 (8.98)			
C-SSRS score^e^					
Baseline	17.31 (2.56)	17.37 (3.08)	3.35 (1.01 to 5.69)	*F*_4,55_ = 2.56	.048
Day 1	13.89 (5.47)	17.00 (2.98)			
Day 2	11.36 (7.00)	15.17 (4.36)			
Day 3	8.29 (7.47)	13.28 (6.16)			
Day 4	7.71 (7.89)	13.24 (6.94)			
BDI score^f^					
Baseline	40.48 (7.79)	41.27 (7.85)	6.73 (0.66 to 12.79)	*F*_4,55_ = 2.23	.08
Day 1	27.75 (14.50)	37.72 (9.88)			
Day 2	26.61 (15.93)	35.59 (10.56)			
Day 3	24.54 (16.95)	32.00 (12.83)			
Day 4	22.07 (17.37)	30.76 (14.08)			
MADRS score^g^					
Baseline	30.38 (4.75)	30.43 (4.62)	3.13 (0.15 to 6.10)	*F*_4,55_ = 1.68	.17
Day 1	20.04 (8.03)	22.86 (5.67)			
Day 2	16.11 (7.55)	20.52 (5.75)			
Day 3	13.61 (8.68)	17.55 (6.14)			
Day 4	10.86 (7.64)	15.90 (7.41)			
HAMA score^h^					
Baseline	13.83 (4.94)	13.80 (5.83)	1.64 (−0.49 to 3.77)	*F*_4,55_ = 0.86	.50
Day 1	8.93 (4.71)	11.17 (5.13)			
Day 2	6.50 (4.22)	8.97 (5.18)			
Day 3	6.18 (4.33)	7.90 (4.80)			
Day 4	5.11 (4.23)	7.07 (4.89)			
BHS score^i^					
Baseline	16.00 (3.26)	16.40 (2.76)	2.76 (0.72 to 4.80)	*F*_4,55_ = 4.09	.006
Day 1	13.32 (4.80)	16.90 (2.26)			
Day 2	12.96 (5.80)	16.66 (2.26)			
Day 3	12.50 (6.32)	15.55 (3.42)			
Day 4	10.93 (6.73)	15.17 (3.70)			
Change in score from baseline to 1 mo					
BSI^d^	10.17 (9.06)	7.43 (10.47)	2.20 (−0.49 to 4.90)	*F*_1,47_ = 1.02	.32
BDI^f^	16.83 (17.01)	9.91 (11.65)	4.38 (−1.22 to 9.99)	*F*_1,44_ = 2.81	.10
BHS^i^	5.00 (5.45)	1.39 (3.46)	2.32 (0.52 to 4.12)	*F*_1,45_ = 8.26	.006

Abbreviations: BDI, Beck Depression Inventory; BHS, Beck Hopelessness Scale; BSI, Beck Scale for Suicide Ideation; C-SSRS, Columbia-Suicide Severity Rating Scale; HAMA, Hamilton Anxiety Rating Scale; MADRS, Montgomery-Åsberg Depression Rating Scale.

^a^ Changes were over the 4-day intervention.

^b^ Changes in scale score were the estimated marginal means from linear mixed-effects models. The differences were calculated based on the estimated marginal means.

^c^ Time × group interaction of the linear mixed-effects model.

^d^ BSI score range, 0 to 38, with higher scores indicating more severe symptoms.

^e^ C-SSRS score range, 0 to 25, with higher scores indicating more severe symptoms.

^f^ BDI score range, 0 to 63, with higher scores indicating more severe symptoms.

^g^ MADRS score range, 0 to 60, with higher scores indicating more severe symptoms.

^h^ HAMA score range, 0 to 56, with higher scores indicating more severe symptoms.

^i^ BHS score range, 0 to 20, with higher scores indicating more severe symptoms.

### Secondary Outcomes

Over the 4-day intervention, the dual-site aiTBS demonstrated significantly lower mean BSI scores compared with the single-site group on each day (difference: day 1, 5.20 points [95% CI, 2.19-8.20 points]; day 2, 6.35 points [95% CI, 2.50-10.21 points]; day 3, 5.50 points [95% CI, 0.79-0.21 points]; day 4, 5.47 points [95% CI, 0.82-10.12 points]) (Table 2 and Figure 2A). This effect was corroborated by a sensitivity analysis using the mean C-SSRS score (difference: day 1, 2.93 points [95% CI, 0.56-5.30 points]; day 2, 3.65 points [95% CI, 0.51-6.79 points]; day 3, 4.80 points [95% CI, 1.10-8.50 points]; day 4, 5.31 points [95% CI, 1.27-9.34 points]) (eFigure 1 in Supplement 2). For depressive symptoms, a significant between-group difference in mean MADRS score was found on days 2 and 4 (difference: day 1, 2.62 points [95% CI, −1.12 to 6.36 points]; day 2, 4.27 points [95% CI, 0.65-7.88 points]; day 3, 3.81 points [95% CI, −0.24 to 7.87 points]; day 4, 4.87 points [95% CI, 0.79-8.95 points]) (eFigure 1 in Supplement 2); sensitivity analysis using the BDI showed significantly lower mean scores in the dual-site group on the first 2 days (difference: day 1, 9.42 points [95% CI, 2.69-16.16 points]; day 2, 8.48 points [95% CI, 1.16-15.80 points]; day 3, 6.97 points [95% CI, −1.17 to 15.11 points]; day 4, 7.98 points [95% CI, −0.60 to 16.57 points]) (Figure 2B). Mean BHS scores were consistently and significantly lower in the dual-site than single-site group across all daily assessments (difference: day 1, 3.27 points [95% CI, 1.21-5.33 points]; day 2, 3.40 points [95% CI, 1.00-5.79 points]; day 3, 2.81 points [95% CI, 0.06-5.56 points]; day 4, 3.92 points [95% CI, 0.96-6.88] points) (Figure 2C). HAMA scores showed a significant main effect of time (*F*_4,55_ = 30.40; *P* < .001), while no significant group (*F*_1,56_ = 2.37; *P* = .13) or time × group interaction (*F*_4,55_ = 0.86; *P* = .50) effects were detected (eFigure 1 in Supplement 2).

At the 1-month follow-up, the linear mixed-effects model indicated a significant main effect of time on both BSI and BDI scores (BSI: *F*_1,47_ = 37.98; BDI: *F*_1,44_ = 38.78; both *P* < .001), with no significant group effects (BSI: *F*_1,46_ = 2.72; *P* = .11; BDI: *F*_1,50_ = 2.47; *P* = .12) or group × time interaction effects (BSI: *F*_1,47_ = 1.02; *P* = .32; BDI: *F*_1,44_ = 2.81; *P* = .10) (Table 2 and Figure 2A and B). Significant effects of group (*F*_1,52_ = 6.55; *P* = .01), time (*F*_1,45_ = 22.78; *P* < .001), and group × time interaction (*F*_1,45_ = 8.26; *P* = .006) were observed for the BHS. Post hoc tests revealed that the dual-site group had significantly greater reduction in mean BHS scores than the single-site group at 1 month (difference, 3.84 points [95% CI, 1.27-6.42 points]; *t* = 2.99; *P* = .004; Cohen *d*, 0.78 [95% CI, 0.25-1.31]) (Figure 2C).

Following the 4-day intervention, for suicidal ideation there were no significant differences among the dual-site vs single-site patients in rates of response (17 [59%] vs 11 [37%]; χ^2^ = 2.85; *P* = .09) or remission (14 [48%] vs 9 [30%]; χ^2^ = 2.07; *P* = .15). In contrast, for depression, response and remission rates were significantly higher in the dual-site group (response: 21 [72%] vs 13 [43%]; χ^2^ = 5.11; *P* = .02; remission: 13 [45%] vs 6 [20%]; χ^2^ = 4.16; *P* = .04) (Table 3).

**Table 3.** Response and Remission Rates of Suicidal Ideation and Depression After Treatment Across Groups

Outcome	Patient group, No. (%)		OR (95% CI)^a^	χ^2^ test	*P* value
	Dual-site (n = 29)	Single-site (n = 30)			
Suicidal ideation^b^					
Response	17 (59)	11 (37)	2.45 (0.86-6. 98)	2.85	.09
Remission	14 (48)	9 (30)	2.18 (0.75-6.34)	2.07	.15
Depression^c^					
Response	21 (72)	13 (43)	3.43 (1.16-10.19)	5.11	.02
Remission	13 (45)	6 (20)	3.25 (1.02-10.32)	4.16	.04

Abbreviation: OR, odds ratio.

^a^ Calculated by logistic regression.

^b^ Assessed by the Beck Scale for Suicide Ideation (score range, 0 to 38, with higher scores indicating more severe symptoms).

^c^ Assessed by Montgomery-Åsberg Depression Rating Scale (score range, 0 to 60, with higher scores indicating more severe symptoms).

Correlation analyses demonstrated that decreases in BSI scores were positively correlated with concurrent decreases in both BDI and BHS scores at day 4 (BDI: *r* = 0.79 [95% CI, 0.66-0.87]; *P* < .001; BHS: *r* = 0.64 [95% CI, 0.46-0.77]; *P* < .001) and at the 1-month follow-up (BDI: *r* = 0.74 [95% CI, 0.60-0.84]; *P* < .001; BHS: *r* = 0.54 [95% CI, 0.32-0.67]; *P* < .001) (eFigure 2 in Supplement 2). Furthermore, the preintervention-to-postintervention reduction in C-SSRS scores was strongly correlated with the reduction in BSI scores (*r* = 0.83 [95% CI, 0.73-0.90]; *P* < .001), and the reduction in MADRS scores was correlated with the reduction in BDI scores (*r* = 0.73 [95% CI, 0.58-0.83]; *P* < .001).

### Adverse Events and Safety

The most common adverse events, calculated as events among all daily treatment sessions in both groups and summed across the 4 days of the intervention, were pain at either of the 2 stimulation sites (14 events in 456 exposures [3%]) and dizziness (10 events in 228 sessions [4%]), with no significant difference between the dual- and single-site groups (eTable in Supplement 2). All adverse events were well tolerated and gradually disappeared within approximately 30 minutes after cessation of treatment. No serious adverse events were reported.

## Discussion

This randomized clinical trial provided initial evidence of the efficacy and safety of dual-site aiTBS targeting the left DLPFC and cerebellum for rapidly reducing suicidal ideation in adolescents with MDD. The dual-site group demonstrated significantly greater reduction in suicidal ideation than the single-site group after the 4-day treatment, with therapeutic benefits evident from the first day of the aiTBS intervention. Concurrent superior reductions in depressive symptoms were also observed. The aiTBS treatment was well tolerated without any serious adverse events, indicating its feasibility.

To enhance antidepressant efficacy beyond standard left DLPFC stimulation, dual-site rTMS protocols have been explored.^30,31^ Previous studies have primarily tested combinations within the prefrontal cortex (eg, bilateral DLPFC or left DLPFC with right orbitofrontal cortex), but results have been mixed regarding their superiority over left DLPFC alone for depressive symptoms.^31,32,33,34,35^ Furthermore, while bilateral prefrontal stimulation showed promise for reducing suicidal ideation compared with sham stimulation, it has not consistently outperformed standard left DLPFC stimulation, curtailing its clinical appeal.^35,36^ In the present study, we found that dual-site aiTBS to the left DLPFC and left cerebellum produced a more rapid onset of action and superior antisuicidal efficacy in adolescents with MDD compared with single-site aiTBS to only the left DLPFC. The results suggest that strategizing neuromodulation around the cerebellar-cerebral network may represent a more viable therapeutic approach than focusing solely on the prefrontal region. This aligns with evidence that MDD is a disorder of distributed brain networks, among which the cerebellar-cerebral circuits have been strongly implicated, rather than of a single dysfunctional brain region.^37,38^ Recently, the modulation of symptom-specific neural circuits with rTMS has been shown to improve clinical outcomes.^39^ Critically, the prefrontal-cerebellar circuit is not only implicated in the pathophysiology of MDD^14,40^ but is also associated with suicidality.^21,41^ Collectively, these findings support that the cerebellum may constitute a novel target to enhance the rapid-onset antisuicidal efficacy of standard left DLPFC aiTBS in adolescents with MDD.

Although standard iTBS is well tolerated, to our knowledge it has not been proven effective beyond placebo in adolescents with MDD,^42,43^ whereas aiTBS has shown promising efficacy against depressive symptoms, anxiety, and nonsuicidal self-injury. For instance, Liu et al^44^ reported that an aiTBS regimen (5 daily sessions of 1800-pulse iTBS to the left DLPFC over 10 days) yielded significantly greater improvements in depression and anxiety than sham treatment in adolescents with non–treatment-resistant MDD, with benefits lasting for 1 month. Furthermore, Qin et al^45^ revealed that a 5-day regimen of daily 1800-pulse iTBS sessions targeting the left DLPFC decreased non–suicidal self-injury behaviors in adolescents with MDD. Similarly, our 4-day aiTBS regimen (5 daily 600-pulse sessions to the left DLPFC) produced rapid reductions in suicidal ideation, depressive symptoms, and anxiety. However, further validation is required due to the lack of a dual-site sham control group in our study. Nevertheless, aiTBS targeting the cerebellum alongside the DLPFC produced a more rapid and pronounced antisuicidal effect in adolescents with moderate-to-severe MDD vs DLPFC stimulation alone. Compared with SNT, our dual-site regimen achieved a shorter treatment duration, lower total pulse number, and simplified targeting, underscoring its high clinical utility and accessibility. Overall, the present study introduced a novel and highly efficient dual-site aiTBS paradigm. By achieving rapid antisuicidal effects with minimal pulses and treatment time, this protocol represents a promising strategy for clinical translation for adolescents with MDD.

### Limitations

This study has several limitations. First, given the single-center design and limited sample size, our findings necessitate validation in larger, multisite trials to confirm both efficacy and generalizability. Second, the study did not include a dual-site sham group. This design choice was made because the primary outcome was to evaluate a dual-target strategy in which the cerebellum was investigated as a synergistic target to enhance the efficacy of left DLPFC aiTBS. Furthermore, for participants with moderate to severe suicidal ideation (BSI score ≥12), a sham intervention was considered ethically inappropriate. Third, while ethically imperative for patients at high suicide risk, the necessary use of concomitant pharmacotherapy is a limitation. Even with medication held constant, the aiTBS outcomes might reflect an interaction with pharmacotherapy rather than its stand-alone efficacy.

## Conclusions

In this randomized clinical trial of 59 adolescents with MDD and suicidal ideation, dual-site aiTBS targeting the left DLPFC and left cerebellum was a more effective rapid-onset treatment for suicidal ideation than single-site aiTBS targeting the left DLPFC alone, and the treatment was well tolerated. Future research should investigate the mechanistic underpinnings of dual-site aiTBS and optimize the stimulation parameters to maximize therapeutic outcomes.

## Notes

Supplement 1.
Trial Protocol

Supplement 2. **eMethods.** Clinical Assessments and Outcomes **eTable.** Incidence of Adverse Events Across Groups **eFigure 1.** Clinical Secondary Outcomes of aiTBS Treatment **eFigure 2.** Relationships Among Changes in Suicidal Ideation, Depression, and Hopelessness Scores

Supplement 3.
Data Sharing Statement

## References

[1] Shain B; COMMITTEE ON ADOLESCENCE. Suicide and suicide attempts in adolescents. Pediatrics. 2016;138(1):e20161420. doi:10.1542/peds.2016-1420 27354459

[2] Strahlman MT, Thomas PB, Hunt ET, Mantey DS. Parental monitoring and adolescent suicidality: exploring differences by sex in the 2021 national survey on drug use and health. J Affect Disord. 2025;381:9-15. doi:10.1016/j.jad.2025.03.170 40187423

[3] Walter G. Nessun dorma (“none shall sleep”)… at least not before we digest treatment of adolescent suicide attempters (TASA). J Am Acad Child Adolesc Psychiatry. 2009;48(10):977-978. doi:10.1097/CHI.0b013e3181b45098 20854766

[4] Keyes KM, Platt JM. Annual Research Review: sex, gender, and internalizing conditions among adolescents in the 21st century - trends, causes, consequences. J Child Psychol Psychiatry. 2024;65(4):384-407. doi:10.1111/jcpp.13864 37458091 PMC12341061

[5] Cole EJ, Phillips AL, Bentzley BS, . Stanford neuromodulation therapy (SNT): a double-blind randomized controlled trial. Am J Psychiatry. 2022;179(2):132-141. doi:10.1176/appi.ajp.2021.20101429 34711062

[6] Li B, Zhao N, Tang N, . Targeting suicidal ideation in major depressive disorder with MRI-navigated Stanford accelerated intelligent neuromodulation therapy. Transl Psychiatry. 2024;14(1):21. doi:10.1038/s41398-023-02707-9 38199983 PMC10781692

[7] Desmyter S, Duprat R, Baeken C, Bijttebier S, van Heeringen K. The acute effects of accelerated repetitive Transcranial Magnetic Stimulation on suicide risk in unipolar depression: preliminary results. Psychiatr Danub. 2014;26(suppl 1):48-52. 25413512

[8] Desmyter S, Duprat R, Baeken C, Van Autreve S, Audenaert K, van Heeringen K. Accelerated intermittent theta burst stimulation for suicide risk in therapy-resistant depressed patients: a randomized, sham-controlled trial. Front Hum Neurosci. 2016;10:480. doi:10.3389/fnhum.2016.00480 27729854 PMC5037167

[9] Baeken C, Wu GR, van Heeringen K. Placebo aiTBS attenuates suicidal ideation and frontopolar cortical perfusion in major depression. Transl Psychiatry. 2019;9(1):38. doi:10.1038/s41398-019-0377-x 30696807 PMC6351528

[10] Huang D, Zhong S, Song X, Zhang R, Lai S, Jia Y. Effect of novel accelerated intermittent theta burst stimulation on suicidal ideation in adolescent patients with major depressive episode: a randomised clinical trial. Gen Psychiatr. 2024;37(2):e101394. doi:10.1136/gpsych-2023-101394 38665940 PMC11043680

[11] Azevedo FA, Carvalho LR, Grinberg LT, . Equal numbers of neuronal and nonneuronal cells make the human brain an isometrically scaled-up primate brain. J Comp Neurol. 2009;513(5):532-541. doi:10.1002/cne.21974 19226510

[12] Herculano-Houzel S, Catania K, Manger PR, Kaas JH. Mammalian brains are made of these: a dataset of the numbers and densities of neuronal and nonneuronal cells in the brain of Glires, Primates, Scandentia, Eulipotyphlans, Afrotherians and Artiodactyls, and their relationship with body mass. Brain Behav Evol. 2015;86(3-4):145-163. doi:10.1159/000437413 26418466

[13] Strick PL, Dum RP, Fiez JA. Cerebellum and nonmotor function. Annu Rev Neurosci. 2009;32:413-434. doi:10.1146/annurev.neuro.31.060407.125606 19555291

[14] Wang X, Xia J, Wang W, . Disrupted functional connectivity of the cerebellum with default mode and frontoparietal networks in young adults with major depressive disorder. Psychiatry Res. 2023;324:115192. doi:10.1016/j.psychres.2023.115192 37054552

[15] Wang L, Zhao P, Zhang J, . Functional connectivity between the cerebellar vermis and cerebrum distinguishes early treatment response for major depressive episodes in adolescents. J Affect Disord. 2023;339:256-263. doi:10.1016/j.jad.2023.07.054 37437740

[16] Sankar A, Scheinost D, Goldman DA, . Graph theory analysis of whole brain functional connectivity to assess disturbances associated with suicide attempts in bipolar disorder. Transl Psychiatry. 2022;12(1):7. doi:10.1038/s41398-021-01767-z 35013103 PMC8748935

[17] Hwang JP, Lee TW, Tsai SJ, . Cortical and subcortical abnormalities in late-onset depression with history of suicide attempts investigated with MRI and voxel-based morphometry. J Geriatr Psychiatry Neurol. 2010;23(3):171-184. doi:10.1177/0891988710363713 20430976

[18] Amen DG, Prunella JR, Fallon JH, Amen B, Hanks C. A comparative analysis of completed suicide using high resolution brain SPECT imaging. J Neuropsychiatry Clin Neurosci. 2009;21(4):430-439. doi:10.1176/jnp.2009.21.4.430 19996252

[19] Johnston JAY, Wang F, Liu J, . Multimodal neuroimaging of frontolimbic structure and function associated with suicide attempts in adolescents and young adults with bipolar disorder. Am J Psychiatry. 2017;174(7):667-675. doi:10.1176/appi.ajp.2016.15050652 28135845 PMC5939580

[20] Li W, Wang C, Lan X, . Variability and concordance among indices of brain activity in major depressive disorder with suicidal ideation: A temporal dynamics resting-state fMRI analysis. J Affect Disord. 2022;319:70-78. doi:10.1016/j.jad.2022.08.122 36075401

[21] Reis JV, Vieira R, Portugal-Nunes C, . Suicidal ideation is associated with reduced functional connectivity and white matter integrity in drug-naïve patients with major depression. Front Psychiatry. 2022;13:838111. doi:10.3389/fpsyt.2022.838111 35386522 PMC8978893

[22] Sherrin T, Heng KY, Zhu YZ, Tang YM, Lau G, Tan CH. Cholecystokinin-B receptor gene expression in cerebellum, pre-frontal cortex and cingulate gyrus and its association with suicide. Neurosci Lett. 2004;357(2):107-110. doi:10.1016/j.neulet.2003.11.072 15036586

[23] Shunkai L, Chen P, Zhong S, . Alterations of insular dynamic functional connectivity and psychological characteristics in unmedicated bipolar depression patients with a recent suicide attempt. Psychol Med. 2023;53(9):3837-3848. doi:10.1017/S0033291722000484 35257645

[24] Richieri R, Verger A, Boyer L, . Predictive value of dorso-lateral prefrontal connectivity for rTMS response in treatment-resistant depression: A brain perfusion SPECT study. Brain Stimul. 2018;11(5):1093-1097. doi:10.1016/j.brs.2018.05.010 29802071

[25] World Medical Association. World Medical Association Declaration of Helsinki: ethical principles for medical research involving human subjects. JAMA. 2013;310(20):2191-2194. doi:10.1001/jama.2013.281053 24141714

[26] Hardwick RM, Lesage E, Miall RC. Cerebellar transcranial magnetic stimulation: the role of coil geometry and tissue depth. Brain Stimul. 2014;7(5):643-649. doi:10.1016/j.brs.2014.04.009 24924734 PMC4180011

[27] Young RC, Biggs JT, Ziegler VE, Meyer DA. A rating scale for mania: reliability, validity and sensitivity. Br J Psychiatry. 1978;133:429-435. doi:10.1192/bjp.133.5.429 728692

[28] Zhao H, Jiang C, Zhao M, . Comparisons of accelerated continuous and intermittent theta burst stimulation for treatment-resistant depression and suicidal ideation. Biol Psychiatry. 2024;96(1):26-33. doi:10.1016/j.biopsych.2023.12.013 38142717

[29] Frank E, Prien RF, Jarrett RB, . Conceptualization and rationale for consensus definitions of terms in major depressive disorder. Remission, recovery, relapse, and recurrence. Arch Gen Psychiatry. 1991;48(9):851-855. doi:10.1001/archpsyc.1991.01810330075011 1929776

[30] Elnazali M, Veerakumar A, Blair M, . Unilateral and bilateral theta burst stimulation for treatment-resistant depression: Follow up on a naturalistic observation study. J Psychiatr Res. 2024;180:387-393. doi:10.1016/j.jpsychires.2024.10.031 39531945

[31] Cui H, Ding H, Hu L, Zhao Y, Shu Y, Voon V. A novel dual-site OFC-dlPFC accelerated repetitive transcranial magnetic stimulation for depression: a pilot randomized controlled study. Psychol Med. 2024;54(14):3849-3862. doi:10.1017/S0033291724002289 39440449 PMC11578911

[32] Liu J, Shu Y, Wu G, Hu L, Cui H. A neuroimaging study of brain activity alterations in treatment-resistant depression after a dual target accelerated transcranial magnetic stimulation. Front Psychiatry. 2024;14:1321660. doi:10.3389/fpsyt.2023.1321660 38288056 PMC10822961

[33] Joseph JT, Jammigumpula A, Vaidyanathan S, Praharaj SK. Sequential bilateral repetitive transcranial magnetic stimulation in depression: Targeting right orbitofrontal cortex and left dorsolateral prefrontal cortex in nonresponders. Asian J Psychiatr. 2025;105:104392. doi:10.1016/j.ajp.2025.104392 39947103

[34] Asgharian Asl F, Abbaszade S, Derakhshani H, Vaghef L, Asgharian Asl A. Unilateral vs. bilateral DLPFC rTMS: comparative effects on depression, visual-spatial memory, inhibitory control and cognitive flexibility in major depressive disorder. Front Psychiatry. 2024;15:1400414. doi:10.3389/fpsyt.2024.1400414 39290299 PMC11405187

[35] Serafini G, Canepa G, Aguglia A, . Effects of repetitive transcranial magnetic stimulation on suicidal behavior: A systematic review. Prog Neuropsychopharmacol Biol Psychiatry. 2021;105:109981. doi:10.1016/j.pnpbp.2020.109981 32485190

[36] Wei Y, Zhai W, Tang X, . Accelerated bilateral theta burst stimulation for suicidal ideation in depression. Brain Stimul. 2025;18(5):1490-1492. doi:10.1016/j.brs.2025.08.001 40782848

[37] Dai P, Huang K, Shi Y, . Effective connectivity between the cerebellum and fronto-temporal regions correctly classify major depressive disorder: fMRI study using a multi-site dataset. J Affect Disord. 2025;390:119783. doi:10.1016/j.jad.2025.119783 40609648

[38] Zhu DM, Yang Y, Zhang Y, . Cerebellar-cerebral dynamic functional connectivity alterations in major depressive disorder. J Affect Disord. 2020;275:319-328. doi:10.1016/j.jad.2020.06.062 32734925

[39] Siddiqi SH, Fox MD. Targeting symptom-specific networks with transcranial magnetic stimulation. Biol Psychiatry. 2024;95(6):502-509. doi:10.1016/j.biopsych.2023.11.011 37979642

[40] Wu Y, Jiang W, Chen M, . Functional connectivity of the default mode network subsystems alterations in suicide attempters with major depressive disorder. Asian J Psychiatr. 2025;107:104456. doi:10.1016/j.ajp.2025.104456 40158274

[41] Deng J, Zhang M, Chen G, . Exploring neural changes associated with suicidal ideation and attempts in major depressive disorder: A multimodal study. Brain Res Bull. 2025;225:111336. doi:10.1016/j.brainresbull.2025.111336 40222622

[42] Wang X, Zhang Z, Li Y, Gao Y, Cao Q. Efficacy and safety of transcranial magnetic stimulation in the treatment of children, adolescents and young adults with depression: A meta-analysis of randomized controlled trials. J Affect Disord. 2026;392:120132. doi:10.1016/j.jad.2025.120132 40865772

[43] Zhang M, Li W, Ye Y, Hu Z, Zhou Y, Ning Y. Efficacy and safety of intermittent theta burst stimulation on adolescents and young adults with major depressive disorder: A randomized, double blinded, controlled trial. J Affect Disord. 2024;350:214-221. doi:10.1016/j.jad.2024.01.025 38199406

[44] Liu X, Peng Z, Cheng F, . Efficacy and safety of accelerated intermittent theta-burst stimulation for adolescents with major depressive disorder: a randomized, double-blind, sham-controlled study. Biol Psychiatry. 2026;99(3):218-226. doi:10.1016/j.biopsych.2025.07.018 40738417

[45] Qin Y, Chen H, Liu F, . Intermittent theta burst stimulation for non-suicidal self-injury in adolescents with major depressive disorder: a randomized, sham-controlled trial. Mol Psychiatry. 2026;31(2):860-868. doi:10.1038/s41380-025-03183-x 40883451 PMC12815678

