# Supplementary material for: Dual-Site aiTBS for Suicidal Ideation in Adolescents With Major Depressive Disorder: A Randomized Clinical Trial
Source: JAMA Netw Open. 2026 May 19;9(5):e2613178. doi: 10.1001/jamanetworkopen.2026.13178 (PMC13187876; doi:10.1001/jamanetworkopen.2026.13178)
Supplement: Supplement 1. — Trial Protocol [file jamanetwopen-e2613178-s001.pdf]

Final Research Protocol

Title of study:

DLPFC-cerebellum dual-site aiTBS for suicidal ideation in adolescents with major depressive disorder: A randomized clinical trial

Research team:

Authors: Dong Huang[1, #], Rongxu Zhang[1, #], Shunkai Lai[1, #], Xiaojie Ye[1], Munila·Abula[1], Xiaodong Song[1], Peiying Cao[1], Yiliang Zhang[1], Jianzhao Zhang[1], Shuming Zhong[1, *], Yanbin Jia[1, *]

1 Department of Psychiatry, First Affiliated Hospital of Jinan University, Guangzhou 510630, China

# Dong Huang, Rongxu Zhang and Shunkai Lai contributed equally to this work and should be considered co-first authors.

*** Correspondence:**

Shuming Zhong, Department of Psychiatry, First Affiliated Hospital of Jinan University, Guangzhou 510630, China. Email: Shuming19882006@126.com

Yanbin Jia, Department of Psychiatry, First Affiliated Hospital of Jinan University, Guangzhou 510630, China. Email: yanbinjia2006@163.com

Contents of the trial protocol

1 Introduction 1

2 Objective 1

3 Hypotheses 1

4 Study design 1

5 Statistical Analysis 4

6 Ethical considerations 4

7 Reference 6

1 Introduction

Suicide represents a leading cause of mortality among adolescents, and major depressive disorder (MDD) is a principal contributing condition[1]. The prevalence of suicidal ideation and attempts is markedly elevated in adolescents with MDD compared to the general population[2,3], establishing an urgent need for effective interventions capable of rapidly mitigating suicide risk.

Current first-line treatments of MDD, including pharmacotherapy and psychotherapy, demonstrate limited and delayed efficacy in adolescents. Selective serotonin reuptake inhibitors may also carry an initial risk of increased suicidality. These limitations have spurred interest in neuromodulation approaches. Repetitive transcranial magnetic stimulation (rTMS) is a non-invasive brain stimulation technique with emerging evidence for treating adolescent MDD[4]. A patterned form of rTMS, intermittent theta burst stimulation (iTBS), delivers therapeutic doses in a fraction of the time required for standard rTMS, thereby enhancing practicality[5].

Further intensifying the treatment protocol, accelerated iTBS (aiTBS) administers multiple daily sessions over a short period (e.g., 20 sessions over 4 days). This accelerated schedule has been shown to induce rapid antidepressant and anti-suicidal effects in adults[6-8], but its efficacy and safety in adolescents remain less established.

The cerebellum has gained recognition as a key node in the neurocircuitry of depression and suicide, beyond its traditional role in motor coordination. Evidence from neuroimaging and postmortem studies indicates structural, functional, and metabolic alterations in the cerebellum of individuals with MDD and suicidality[9-14]. Given its dense connectivity to prefrontal regions like the dorsolateral prefrontal cortex (DLPFC)[15]—a primary rTMS target for MDD—the cerebellum presents a compelling secondary target for dual-site stimulation. Modulating this prefrontal-cerebellar circuit is hypothesized to yield superior clinical outcomes compared to DLPFC stimulation alone. Preliminary data from our group supports the feasibility, tolerability, and potential efficacy of a dual-site aiTBS protocol targeting both the left DLPFC and the left cerebellum in adolescents with depression[16].

2 Objective

Accordingly, this study aims to evaluate the efficacy and acceptability of dual-site aiTBS for the rapid reduction of suicidal ideation in adolescents with MDD, using a randomized, double-blind, sham-controlled study.

3 Hypotheses

1) Patients in the dual-site aiTBS group would have a greater reduction in Beck Scale for Suicidal Ideation (BSI) score after treatment than the single-site aiTBS group.
2) Patients in the dual-site aiTBS group would have the same safety as the single-site aiTBS group.

4 Study design

4.1 Study participants

We will conduct a single-site, randomized, double-blind, sham-controlled, two-arm clinical trial. Eligible patients with MDD will be recruited from the First Affiliated Hospital of Jinan University in Guangzhou, China. After screening against the inclusion and exclusion criteria, eligible patients will provide written informed consent and be randomly assigned to one of two intervention groups: the dual-site aiTBS group or the single-site aiTBS group.

(1) Inclusion criteria:

• diagnosed with MDD according to Diagnostic and Statistical Manual of Mental Disorders (Fifth Edition, DSM-5) by two independent psychiatrists;
• aged 12-18 years;
• 24-item Hamilton Depression Rating Scale (HDRS-24) score > 20;
• BSI score ≥ 12;
• Young Mania Rating Scale (YMRS) < 7;
• able to tolerate the treatment;
• agreeing to participate in this study and signed a consent form.

(2) Exclusion criteria:
• comorbid medical or neurological conditions (e.g., epilepsy, brain tumors or trauma);
• a history of neuromodulation therapies (including rTMS, transcranial direct current stimulation, or electroconvulsive therapy) within the past 3 months;
• contraindications such as metal implants.

(3) Criteria for withdrawal:
• non-adherence, defined as refusal of the assigned treatment on ≥ 2 occasions;
• intolerance due to serious adverse effects;
• clinical deterioration requiring a fundamental change in treatment strategy;
• protocol violations, including changes to concomitant medication type or dose during the 4-day intervention.

4.2 Study procedure

(1) Trial visits

Clinical symptom assessments were performed by trained raters blinded to treatment assignments at baseline and daily over the 4-day intervention (using both clinician-rated and self-report scales) and at 1-month follow-up (self-report scales only). Clinical assessments used the following instruments: the HDRS-24 for screening; the Beck Depression Inventory (BDI) and Montgomery-Åsberg Depression Rating Scale (MADRS) for depressive symptoms; the Hamilton Anxiety Rating Scale (HAMA) for anxiety symptoms; the BSI and Columbia-Suicide Severity Rating Scale (C-SSRS) for suicidal ideation; the Beck Hopelessness Scale (BHS) for hopelessness; and the YMRS to monitor for treatment-emergent mood switches.

(2) Protocol for aiTBS

Following randomization and baseline assessment, patients will undergo aiTBS intervention. The aiTBS will be performed using a MagStim Rapid^2 transcranial magnetic stimulator (Magstim Company Ltd.) with a 70-mm air-cooled figure-of-eight coil. The left DLPFC was localized using the 5-cm rule. The left cerebellar target was localized 3 cm lateral and 1 cm inferior to the inion[17]. The resting motor threshold (RMT) was defined as the lowest stimulation intensity required to produce a visible twitch in the relaxed right abductor pollicis brevis muscle in at least 5 of 10 consecutive trials. All stimulation was set at 80% of RMT.

Each iTBS session consisted of 600 pulses delivered over 20 cycles. Each cycle comprised a 2-second train of 10 triple-pulse bursts at 20 Hz, followed by an 8-second intertrain interval, resulting in a total session duration of 3 minutes and 9 seconds[18]. The aiTBS regimen included 5 iTBS sessions per day, separated by 1-hour intervals, over 4 consecutive days, for a total of 12,000 pulses[16]. For sham stimulation, the coil was positioned at a 90° angle to the scalp, with one wing maintaining contact, and was applied to the left cerebellum.

Adverse events (AEs) potentially related to the intervention (e.g., headache, pain, discomfort) were monitored and recorded throughout the study. Participants experiencing serious AEs were withdrawn from the study immediately.

4.3 Randomization and Blinding

Participants were randomly assigned in a 1:1 ratio to one of two treatment groups: (1) the dual-site aiTBS group, which received active aiTBS to both the left DLPFC and the left cerebellum, or (2) the single-site aiTBS group, which received active aiTBS to the left DLPFC and sham stimulation to the left cerebellum. Randomization was based on a computer-generated sequence, prepared by an independent statistician. Allocation was implemented using sequentially numbered, sealed, opaque envelopes. The assignment was executed by an unblinded research coordinator who had no role in clinical assessments, data analysis, or patient care.

To maintain blinding, the patients, outcome assessors, and treating physicians were unaware of the group assignment until the entire study was completed. Only the rTMS operator, who were not involved in outcome assessment, had access to the randomization list. At the end of the follow-up period, participants were asked to guess their assigned treatment group to assess the integrity of the blinding. Subsequently, participants were fully debriefed regarding their actual treatment condition.

4.4 Measurement of Outcomes

(1) Primary Outcome

The primary outcome was the change in BSI scores from baseline to post-intervention (day 4).

(2) Secondary Outcomes

Secondary outcomes included: (1) the daily change in scores on all outcome measures (BSI, BDI, BHS, C-SSRS, MADRS, HAMA) from baseline throughout the 4-day intervention; (2) the change in scores of the self-report scales (BSI, BDI, BHS) from baseline to month 1; (3) response rate at day 4, defined as ≥ 50% reduction in BSI scores for suicidal ideation or MADRS scores for depression; (4) remission rates at day 4, defined as BSI score ≤ 8 for suicidal ideation[19] or MADRS score ≤ 10 for depression[20]; (5) correlation between changes in BSI scores and BDI or BHS scores from baseline to day 4 and to month 1; and (6) incidence of adverse events during the 4-day intervention period.

The flow chart for each patient is shown below (Table 1).

Table 1 Standard protocol items: recommendations for Interventional Trials (SPIRIT) schedule for enrollment, treatment, and assessments.

	STUDY PERIOD							
	Enrolment	Allocation	Post-allocation					Follow-up
TIMEPOINT		0	Baseline	Day 1	Day 2	Day 3	Day 4	Month 1
ENROLMENT								
Eligibility screen	X							
Informed consent	X							
Allocation		X						
INTERVENTIONS:								
Group A				X	X	X	X	
Group B				X	X	X	X	
ASSESSMENTS:								
HDRS-24	X							
YMRS	X		X	X	X	X	X	
MADRS			X	X	X	X	X	
C-SSRS			X	X	X	X	X	
HAMA			X	X	X	X	X	
BSI	X		X	X	X	X	X	X
BDI			X	X	X	X	X	X
BHS			X	X	X	X	X	X
Adverse events				X	X	X	X	

4.5 Sample Size Calculation

The a priori sample size calculation was based on our pilot data indicating a between-group difference in BSI scores that justified a 5-point margin for the present trial. With 80% power and a two-sided α of 0.05, the calculation required 48 participants (24 per group). Allowing for a 20% dropout rate, the target enrollment was set at 58 participants (29 per group).

5 Statistical Analysis

Statistical analyses were performed using IBM SPSS Statistics, Version 25.0. Intention-to-treat analysis was conducted, with missing data estimated by mean interpolation. For the primary outcome and all continuous secondary outcomes, treatment efficacy was tested using linear mixed-effects models. The models included time, group, and the time-by-group interaction as fixed effects, with subject-specific intercepts included as random effects to account for within-participant correlations.

For binary outcomes, such as treatment response, between-group comparisons were performed using the χ^2 test or Fisher exact test, as appropriate. All *P* values were 2-sided, and a *P* value of $< .05$ was defined as statistically significant. Effect sizes were calculated as Cohen's d for continuous outcomes and as odds ratios for binary outcomes.

6 Ethical considerations

6.1 Ethics approval and informed consent

The study protocol was approved by the Medical Research Ethics Committee of the First Affiliated

Hospital of Jinan University. The study was conducted in accordance with the ethical principles of the Declaration of Helsinki. All participants and their legal guardians provided written informed consent prior to enrollment.

Participant safety was actively monitored throughout the study. All participants were instructed to report any adverse events (AEs) spontaneously. Additionally, they were systematically queried about the occurrence of any discomfort or potential AEs during daily visits. Adverse events were assessed by the rTMS operators and clinical assessors. The final decision to withdraw a participant from the study was made by the principal investigator based on the assessed events and predetermined safety criteria.

6.2 Data management

To ensure confidentiality, all collected data were stored in a password-protected electronic file. Access to the database was restricted to authorized members of the research team. Passwords were changed at minimum on a quarterly basis. Upon study completion, the complete dataset was transferred to the principal investigator for secure archiving. Access to the archived data for future use requires formal approval from the principal investigator and the ethics committee.

Reference:

1. Shain B. Suicide and Suicide Attempts in Adolescents. *Pediatrics*. 2016;138(1)doi:10.1542/peds.2016-1420
2. Walter G. Nessun Dorma ("None Shall Sleep")… At least not before we digest Treatment of Adolescent Suicide Attempters (TASA). *J Am Acad Child Adolesc Psychiatry*. 2009;48(10):977-978. doi:10.1097/CHI.0b013e3181b45098
3. Strahlman MT, Thomas PB, Hunt ET, Mantey DS. Parental monitoring and adolescent suicidality: Exploring differences by sex in the 2021 national survey on drug use and health. *J Affect Disord*. 2025;381:9-15. doi:10.1016/j.jad.2025.03.170
4. Narang P, Madigan K, Sarai S, Lippmann S. Is Transcranial Magnetic Stimulation Appropriate For Treating Adolescents with Depression? *Innov Clin Neurosci*. 2019;16(9-10):33-35.
5. Blumberger DM, Vila-Rodriguez F, Thorpe KE, et al. Effectiveness of theta burst versus high-frequency repetitive transcranial magnetic stimulation in patients with depression (THREE-D): a randomised non-inferiority trial. *Lancet*. 2018;391(10131):1683-1692. doi:10.1016/s0140-6736(18)30295-2
6. Desmyter S, Duprat R, Baeken C, Bijttebier S, van Heeringen K. The acute effects of accelerated repetitive Transcranial Magnetic Stimulation on suicide risk in unipolar depression: preliminary results. *Psychiatr Danub*. 2014;26 Suppl 1:48-52.
7. Desmyter S, Duprat R, Baeken C, Van Autreve S, Audenaert K, van Heeringen K. Accelerated Intermittent Theta Burst Stimulation for Suicide Risk in Therapy-Resistant Depressed Patients: A Randomized, Sham-Controlled Trial. *Front Hum Neurosci*. 2016;10:480. doi:10.3389/fnhum.2016.00480
8. Baeken C, Wu GR, van Heeringen K. Placebo aiTBS attenuates suicidal ideation and frontopolar cortical perfusion in major depression. *Transl Psychiatry*. 2019;9(1):38. doi:10.1038/s41398-019-0377-x
9. Sankar A, Scheinost D, Goldman DA, et al. Graph theory analysis of whole brain functional connectivity to assess disturbances associated with suicide attempts in bipolar disorder. *Transl Psychiatry*. 2022;12(1):7. doi:10.1038/s41398-021-01767-z
10. Hwang JP, Lee TW, Tsai SJ, et al. Cortical and subcortical abnormalities in late-onset depression with history of suicide attempts investigated with MRI and voxel-based morphometry. *J Geriatr Psychiatry Neurol*. 2010;23(3):171-84. doi:10.1177/0891988710363713
11. Amen DG, Prunella JR, Fallon JH, Amen B, Hanks C. A comparative analysis of completed suicide using high resolution brain SPECT imaging. *J Neuropsychiatry Clin Neurosci*. 2009;21(4):430-9. doi:10.1176/jnp.2009.21.4.430
12. Johnston JAY, Wang F, Liu J, et al. Multimodal Neuroimaging of Frontolimbic Structure and Function Associated With Suicide Attempts in Adolescents and Young Adults With Bipolar Disorder. *Am J Psychiatry*. 2017;174(7):667-675. doi:10.1176/appi.ajp.2016.15050652
13. Li W, Wang C, Lan X, et al. Variability and concordance among indices of brain activity in major depressive disorder with suicidal ideation: A temporal dynamics resting-state fMRI analysis. *J Affect Disord*. 2022;319:70-78. doi:10.1016/j.jad.2022.08.122
14. Reis JV, Vieira R, Portugal-Nunes C, et al. Suicidal Ideation Is Associated With Reduced Functional Connectivity and White Matter Integrity in Drug-Naïve Patients With Major Depression. *Front Psychiatry*. 2022;13:838111. doi:10.3389/fpsyt.2022.838111

15. Richieri R, Verger A, Boyer L, et al. Predictive value of dorso-lateral prefrontal connectivity for rTMS response in treatment-resistant depression: A brain perfusion SPECT study. *Brain Stimul*. 2018;11(5):1093-1097. doi:10.1016/j.brs.2018.05.010
16. Huang D, Zhong S, Song X, Zhang R, Lai S, Jia Y. Effect of novel accelerated intermittent theta burst stimulation on suicidal ideation in adolescent patients with major depressive episode: a randomised clinical trial. *Gen Psychiatr*. 2024;37(2):e101394. doi:10.1136/gpsych-2023-101394
17. Hardwick RM, Lesage E, Miall RC. Cerebellar transcranial magnetic stimulation: the role of coil geometry and tissue depth. *Brain Stimul*. 2014;7(5):643-9. doi:10.1016/j.brs.2014.04.009
18. Huang YZ, Edwards MJ, Rounis E, Bhatia KP, Rothwell JC. Theta burst stimulation of the human motor cortex. *Neuron*. 2005;45(2):201-6. doi:10.1016/j.neuron.2004.12.033
19. Zhao H, Jiang C, Zhao M, et al. Comparisons of Accelerated Continuous and Intermittent Theta Burst Stimulation for Treatment-Resistant Depression and Suicidal Ideation. *Biol Psychiatry*. 2024;96(1):26-33. doi:10.1016/j.biopsych.2023.12.013
20. Frank E, Prien RF, Jarrett RB, et al. Conceptualization and rationale for consensus definitions of terms in major depressive disorder. Remission, recovery, relapse, and recurrence. *Arch Gen Psychiatry*. 1991;48(9):851-5. doi:10.1001/archpsyc.1991.01810330075011
