# Supplementary material for: Dual-Site aiTBS for Suicidal Ideation in Adolescents With Major Depressive Disorder: A Randomized Clinical Trial
Source: JAMA Netw Open. 2026 May 19;9(5):e2613178. doi: 10.1001/jamanetworkopen.2026.13178 (PMC13187876; doi:10.1001/jamanetworkopen.2026.13178)
Supplement: Supplement 2. — eMethods. Clinical Assessments and Outcomes eTable. Incidence of Adverse Events Across Groups eFigure 1. Clinical Secondary Outcomes of aiTBS Treatment eFigure 2. Relationships Among Changes in Suicidal Ideation, Depression, and Hopelessness Scores [file jamanetwopen-e2613178-s002.pdf]

Supplementary Online Content

Huang D, Zhang R, Lai S, et al. Dual-site aiTBS for suicidal ideation in adolescents with major depressive disorder: a randomized clinical trial. *JAMA Netw Open*. 2026;9(5):e2613178. 10.1001/jamanetworkopen.2026.13178

eMethods. Clinical Assessments and Outcomes

eTable. Incidence of Adverse Events Across Groups

eFigure 1. Clinical Secondary Outcomes of aiTBS Treatment

eFigure 2. Relationships Among Changes in Suicidal Ideation, Depression, and Hopelessness Scores

This supplementary material has been provided by the authors to give readers additional information about their work.

© 2026 Huang D et al. *JAMA Network Open*.

eMethods. Clinical Assessments and Outcomes

Trained research staff, blinded to treatment assignments, conducted all assessments. The Structured Clinical Interview for DSM-5 (SCID-5) was used for psychiatric diagnosis, while the HDRS-24 served as a screening tool. Suicidal ideation was evaluated using both the BSI and the Columbia-Suicide Severity Rating Scale (C-SSRS). Depressive symptom was assessed using the Beck Depression Inventory (BDI) and the Montgomery-Åsberg Depression Rating Scale (MADRS). Anxiety symptom was measured with the Hamilton Anxiety Rating Scale (HAMA), and hopelessness was assessed with the Beck Hopelessness Scale (BHS). The YMRS was evaluated for mood switches emerging from treatment, characterized by a score of 12 or higher.

To comprehensively assess the primary outcome (suicidal ideation) and the primary disease state (major depressive episode), we employed both self-reported (BSI, BDI) and clinician-administered (C-SSRS, MADRS) scales. This dual approach strengthens the reliability and validity of outcome measurement for both constructs. For C-SSRS, only the five-item suicidal ideation subscale was used, with each item rated 1 to 5 (total scores, 0–25); higher scores indicate greater severity of suicidal ideation.

Clinical symptom assessments were performed at baseline and daily over the 4-day intervention and at 1-month follow-up. All measures, including both self-report (BSI, BDI, BHS) and clinician-rated (C-SSRS, MADRS, HAMA) scales, were administered at baseline and daily over the 4-day intervention period. The assessment on day 4 served as the primary post-treatment endpoint. The self-report measures (BSI, BDI, BHS) were subsequently readministered at the 1-month follow-up (±3-day window). The primary outcome was the change in BSI scores from baseline to day 4.

Secondary outcomes included: (1) the daily change from baseline in scores on all outcome measures (BSI, BDI, BHS, C-SSRS, MADRS, HAMA) over the 4-day intervention; (2) the change in scores of the self-report scales (BSI, BDI, BHS) from baseline to month 1; (3) response rate at day 4, defined as ≥ 50% reduction in BSI scores for suicidal ideation or MADRS scores for depression; (4) remission rates at day 4, defined as BSI score ≤ 8 for suicidal ideation or MADRS score ≤ 10 for depression; (5) correlation between changes in BSI scores and BDI or BHS scores from baseline to day 4 and to month 1; and (6) incidence of adverse events during the 4-day intervention period.

The intensive daily assessment schedule was designed to capture rapid changes in clinical states during the acute intervention phase. To manage participant burden and maintain data quality, the following procedures were implemented: (1) all assessments on a given day were conducted in a single session, with breaks offered between scales to prevent fatigue; (2) the order of scale administration was fixed to maintain consistency across participants and days; and (3) the duration of each clinical interview was carefully managed by trained staff to be thorough yet efficient. Participants were fully informed about the time commitment involved during the consent process, and their willingness to comply with this schedule was a criterion for enrollment.

© 2026 Huang D et al. *JAMA Network Open*.

eTable. Incidence of Adverse Events Across Groups

Adverse events	Patient group[a], n (%)		χ^2	*P* value
	Intervention (n = 28)	Control (n = 29)		
Pain at stimulation site (DLPFC)	4 (4)	6 (5)	0.07	.79
Pain at stimulation site (CB)	4 (4)	0 (0)	NA	.06
Dizziness	7 (6)	3 (3)	1.06	.30
Tinnitus	1 (1)	0 (0)	NA	.49

Abbreviations: DLPFC, Dorsolateral Prefrontal Cortex; CB, Cerebellum; NA, not applicable. [a] Adverse events were counted once daily for each participant (maximum: 4 over the 4-day treatment) excluding 2 withdrawals.

© 2026 Huang D et al. *JAMA Network Open*.

eFigure 1. Clinical Secondary Outcomes of aiTBS Treatment

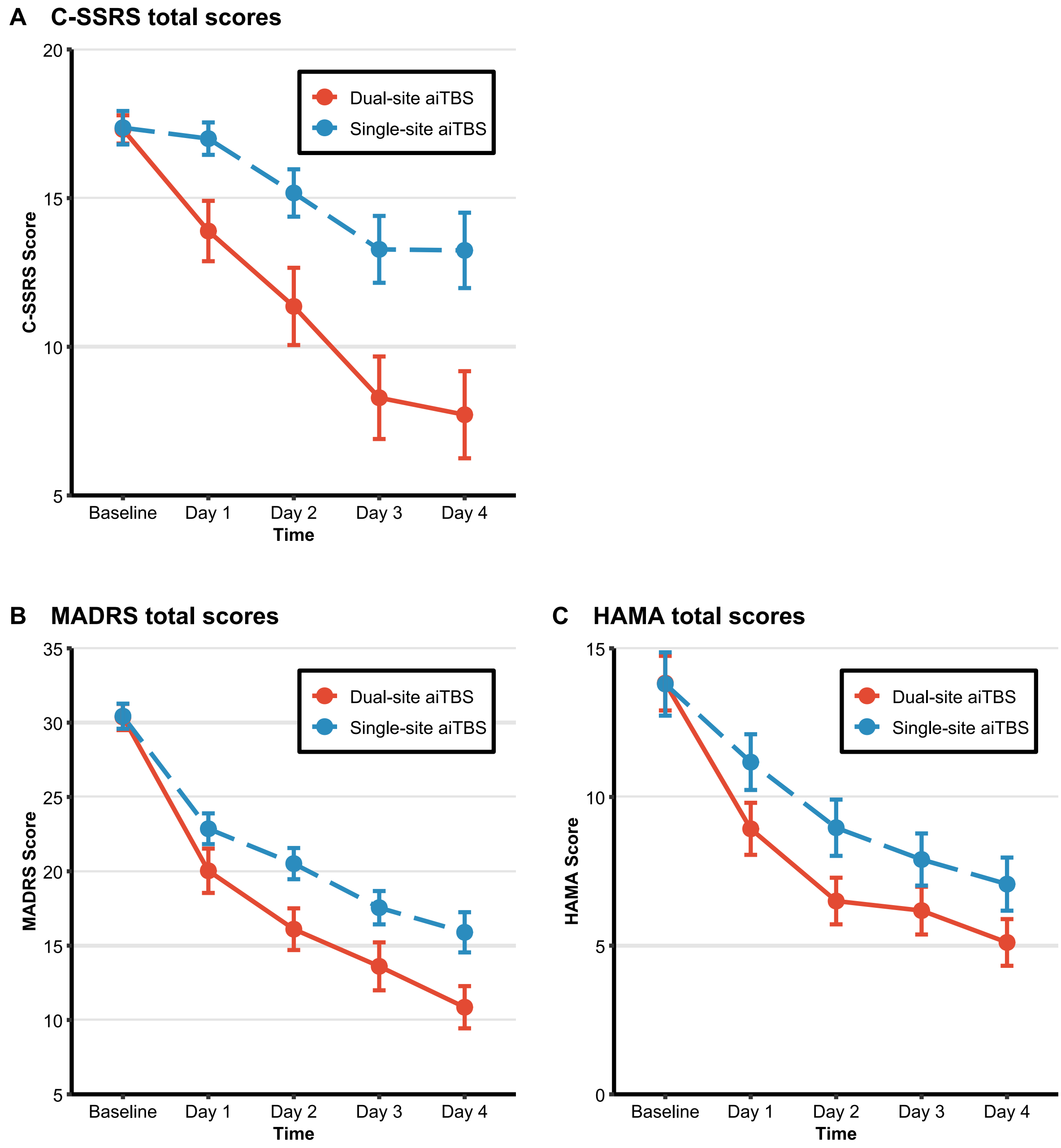

Note: Panel A shows the change in scores on the Columbia-Suicide Severity Rating Scale (C-SSRS). Panel B shows the change in scores on the Montgomery-Åsberg Depression Rating Scale (MADRS). Panel C shows the change in scores on the Hamilton Anxiety Rating Scale (HAMA). aiTBS indicates accelerated intermittent theta burst stimulation. Day 1–4: intervention; Month 1: follow-up. Circles denote means, and error bars denote standard error of the mean.

© 2026 Huang D et al. *JAMA Network Open*.

eFigure 2. Relationships among changes in suicidal ideation, depression, and hopelessness scores

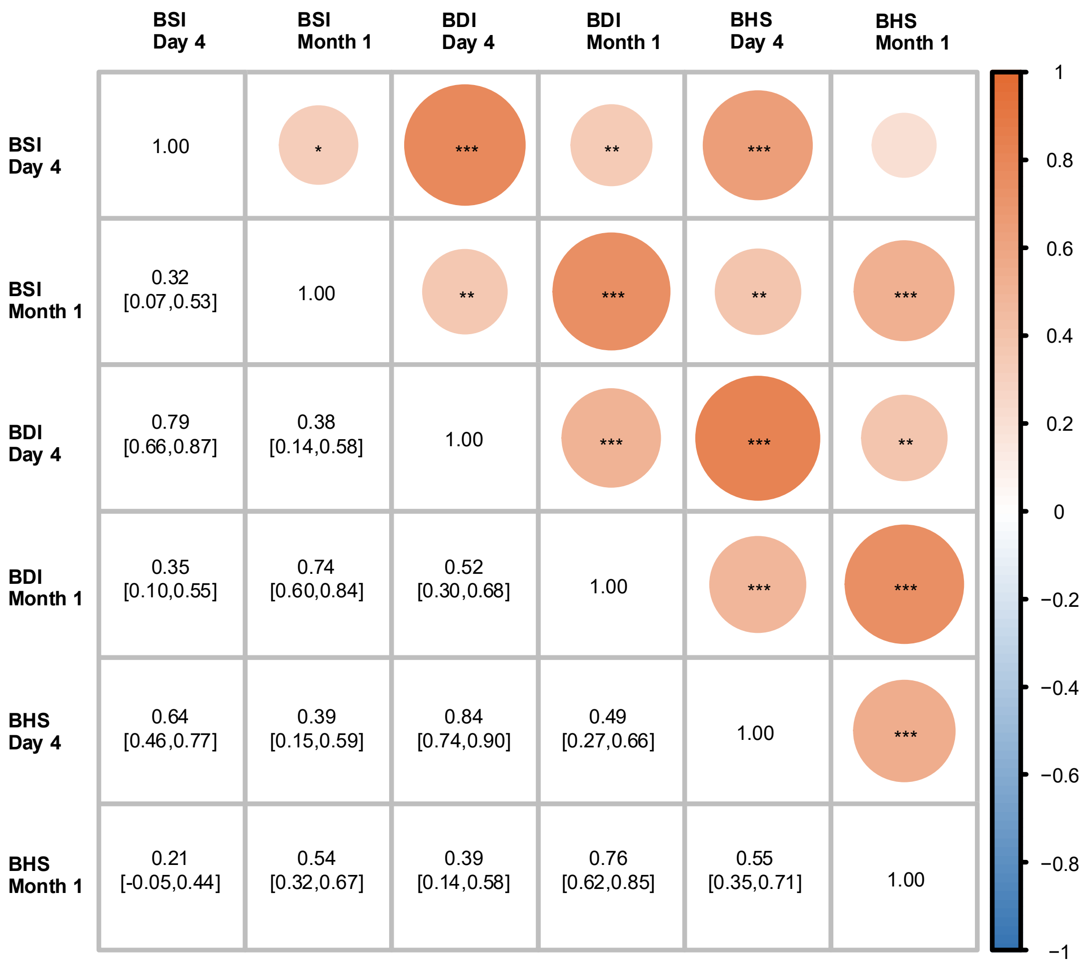

Note: BSI indicates the Beck Scale for Suicidal Ideation. BDI indicates the Beck Depression Inventory. BHS indicates the Beck Hopelessness Scale. Day 4 indicates the change in scale scores from baseline to post-intervention. Month 1 indicates the change in scale scores from baseline to the 1-month follow-up. Correlation coefficients are presented with 95% confidence intervals. * indicates significant relationships. *P < .05; **P < .01; ***P < .001.

© 2026 Huang D et al. *JAMA Network Open*.
