# Supplementary material for: Dual-Site aiTBS for Suicidal Ideation in Adolescents With Major Depressive Disorder: A Randomized Clinical Trial
Source: JAMA Netw Open. 2026 May 19;9(5):e2613178. doi: 10.1001/jamanetworkopen.2026.13178 (PMC13187876; doi:10.1001/jamanetworkopen.2026.13178)
Supplement: Supplement 3. — Data Sharing Statement [file jamanetwopen-e2613178-s003.pdf]

Data Sharing Statement

Huang. Dual-Site aiTBS for Suicidal Ideation in Adolescents With Major Depressive Disorder. *JAMA Netw Open*. Published May 19, 2026. doi:10.1001/jamanetworkopen.2026.13178

Data
Additional Information: Chinese Clinical Trial Registry (ChiCTR);https://www.chictr.org.cn/;ChiCTR2300068954
Data available: Yes
Data types: Other (please specify)
Additional Information: apply to the author if there is a need for relevant data
How to access data: The data that support the findings of this study are available from the corresponding author, Shu-Ming Zhong (Shuming19882006@126.com), upon reasonable request.
When available: With publication

Supporting Documents
Document types: Other (please specify)
Additional Information: Trial protocol in Supplement 1.
How to access documents: See Supplement 1
When available: With publication

Additional Information
Who can access the data: Researchers who provide a methodologically sound proposal that includes a protocol and a statistical analysis plan, and is not in conflict with the investigators'publication plan.
Types of analyses: For a specified purpose.
Mechanisms of data availability: To gain access, data requestors will need to sign a data access agreement.
